# Implementation of written structured feedback into a surgical OSCE

**DOI:** 10.1186/s12909-021-02581-3

**Published:** 2021-04-06

**Authors:** J. Sterz, S. Linßen, M. C. Stefanescu, T. Schreckenbach, L. B. Seifert, M. Ruesseler

**Affiliations:** 1 Department of Trauma, Hand and Reconstructive Surgery, University Hospital Frankfurt, Goethe University, Theodor-Stern-Kai 7, 60590 Frankfurt, Germany; 2 Department of General and Visceral Surgery, University Hospital Frankfurt, Goethe University, Theodor-Stern-Kai 7, 60590 Frankfurt, Germany; 3 Department of Oral, Cranio-Maxillofacial and Facial Plastic Surgery, University Hospital Frankfurt, Goethe University, Theodor-Stern-Kai 7, 60590 Frankfurt, Germany

**Keywords:** Structured written feedback, OSCE, Surgery, Undergraduate medical education

## Abstract

**Background:**

Feedback is an essential element of learning. Despite this, students complain about receiving too little feedback in medical examinations, e.g., in an objective structured clinical examination (OSCE). This study aims to implement a written structured feedback tool for use in OSCEs and to analyse the attitudes of students and examiners towards this kind of feedback.

**Methods:**

The participants were OSCE examiners and third-year medical students.

This prospective study was conducted using a multistage design. In the first step, an unstructured interrogation of the examiners formed the basis for developing a feedback tool, which was evaluated and then adopted in the next steps.

**Results:**

In total, 351 students and 51 examiners participated in this study.

A baseline was created for each category of OSCE station and was supplemented with station-specific items. Each of these items was rated on a three-point scale. In addition to the preformulated answer options, each domain had space for individual comments.

A total of 87.5% of the students and 91.6% of the examiners *agreed* or *rather agreed* that written feedback should continue to be used in upcoming OSCEs.

**Conclusion:**

The implementation of structured, written feedback in a curricular, summative examination is possible, and examiners and students would like the feedback to be constant.

## Background

### Feedback

Feedback is often described as an essential element of learning and education [1–4]. Van de Ridder et al. defined feedback as “specific information about the comparison between a trainee’s observed performance and a standard, given with the intent to improve the trainee’s performance” [5]. Accordingly, the purpose of feedback in medical education is to inform students about the difference between expected learning goals and the performance shown. This is important, as students can use this information to improve their performance and to achieve the defined learning goals more effectively [5, 6].

Despite this, students complain that too little feedback is provided in medical education [7]. Possible reasons for this are the fear of destroying a good relationship with the learner through negative feedback or the lack of time with which to integrate feedback into everyday clinical practice [8]. When feedback files are provided online after assessments, students do not adequately use them; 38% of such feedback files are never opened by students. This number rises to 42% when the assessment marks can be obtained without opening the associated feedback files [9]. Similar results have been found by Henry et al. in 2018. In the context of a simulation-based team-training programme for paediatric residents and nurses, the majority of participants do not seek feedback if it requires increased effort, namely, a personal discussion with one of the tutors with whom participants had scheduled themselves [10]. On the other hand, Juenger et al. demonstrated the importance of feedback to be able to safely assess one’s own performance. The authors were able to demonstrate that 16% of medical students clearly overstate their performance during an internal medicine OSCE [11]. Furthermore, Sinclair and Cleland showed that medical students who achieved lower marks are significantly less likely to seek feedback [12]. Against this background, it is even more important that examinations in medical studies provide valid and useful feedback to medical students that goes beyond a simple grade.

### OSCEs and feedback

OSCEs are a proven and well-studied method for assessing practical skills in medicine. Since their first description by Harden et al. in 1975 [13], OSCEs have been implemented at nearly all medical faculties in Germany [14]. Despite this, there is still disagreement about the best way to integrate feedback into examinations. This disagreement has become even more important, as Harrison et al. stated that OSCEs as “summative assessments have created a powerful culture that is dominated by fear of failure and potential punishment”, which could hinder the use of a summative OSCE as a learning opportunity [15].

One possibility is to implement oral feedback. However, Humphrey-Murto et al. demonstrated that residents remember only 10% of their personal direct oral feedback immediately after an OSCE and that one month later, they are no longer able to repeat concrete aspects of the feedback [16]. It can be assumed that these results are transferable to students’ learning. Furthermore, it seems possible that direct oral feedback in a summative OSCE influences students’ results at the following stations, which must be prevented in an examination that may be crucial to the further course of the study. Therefore, alternatives to direct oral feedback in summative OSCEs are needed. One alternative is written feedback. In 2018, Wardmann et al. showed that students appreciate personal written feedback following an OSCE in dental education [17]. In comparing audio and written feedback during a science laboratory-based core module in which students received feedback on a laboratory report, Morris and Chikwa demonstrated that the way the feedback is provided does not influence the students’ performance in subsequent assessments [18]. However, students have reported that they prefer written feedback, as they find it easier to access such feedback prior to the next set of assessments [18]. Furthermore, Haghani et al. demonstrated that verbal feedback alone is not as effective as verbal feedback combined with written feedback [19].

Junod Perron et al. demonstrated that feedback given by generalist tutors and specialist tutors differs in terms of content [20]. Especially for undergraduate medical students in a curricular setting, it seems necessary to obtain feedback about predefined topics that are important for their further work and that these topics cover the defined learning objectives. Furthermore, Newton et al. demonstrated that, compared to free-text feedback after summative assessments (including assignments such as oral presentations or poster presentations), the use of a structured document defining the domains of the feedback is associated with a significant increase in the quantity of the feedback [21]. Despite all these studies, the best way to incorporate feedback into a summative OSCE has not yet been proven.

Against this background, the present study aimed to create and evaluate a feedback tool that, on the one hand, allows the examiners to provide individualized feedback to the students, and that, on the other hand, ensures that this feedback covers predefined domains, is easy to fulfil during the examination, and can be reviewed by the students after the examination. Furthermore, this study aimed to analyse the attitude of students and examiners towards this kind of feedback.

## Methods

### Study design

The present study followed a prospective design. It was performed according to the ethical principles of the World Medical Association Declaration of Helsinki: Ethical Principles for Medical Research Involving Human Subjects and was reviewed by the ethical committee of the University Hospital of Frankfurt (Johann Wolfgang Goethe University). No further approval was required.

### Participants

The study participants were OSCE undergraduate medical students and examiners at Goethe University in Frankfurt, Germany who were involved in taking or administering the OSCE in surgery during the study period. For both students and examiners, participation in the study was voluntary and revocable at any time.

The OSCE in surgery must be completed by all third-year medical students as part of their curricular surgical training. This summative OSCE is rated by grades from ‘1’, meaning very good, to ‘6’, meaning unsatisfactory. The grades are calculated from the percentage of points achieved. To pass this exam, students must achieve at least 60% of the possible points. Before the present study, these grades were the only feedback that students obtained after completing the OSCE. Prior to taking the OSCE, the students attended two lecture series in surgery and completed a surgical internship consisting of 1 week of training in the surgical skills lab [22] and 2 weeks in surgical wards.

The examiners were surgeons from all surgical disciplines and all stages of professional training. Before they participated in the OSCE, they had to complete a training course.

The OSCE is summative and consists of eight five-minute stations: two evaluating the taking of a patient’s history, two evaluating a physical examination, two evaluating practical skills, e.g., the insertion of an intravenous catheter, and two evaluating obtaining informed consent for a surgical operation.

### Study protocol

**Fig. 1:**
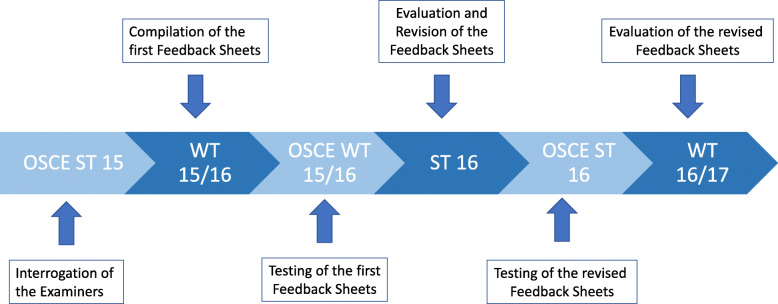
Study design

The study took place over a period of three semesters during 2015 and 2016. The study was conducted using a multistage design that is shown schematically in Fig. 1. To create the feedback tool, a modified nominal group technique was used.

#### Interrogation of the examiners

In the first step, which took place during the OSCE summer term of 2015, 12 examiners with long-term experience (a minimum of five years of experience as OSCE examiners) were asked to write, on a blank sheet, the feedback they would like to give the students. No further instructions regarding the domains of this feedback were provided. To encourage the receipt of individualized information from each examiner, the examiners were instructed neither to compare their notes with each other nor to talk about them.

#### Compilation of the first feedback tool

In the second step, the information collected in the first step was compiled, and related themes were clustered by an expert panel. There were six domains for which the examiners wished to give feedback, and these domains could be identified independently of the content of the individual OSCE station:

- structuring
- time management
- behaviour towards the patient
- manner of speaking
- hygiene issues
- practical execution

Each of these domains was rated on a three-point scale, which was divided into “You performed well in …” , “You could improve in …” , and “You definitely need to improve in …” .

For each of these points, predefined options with further details for improvement were created (e.g., “Give the patient more space to ask questions”, “Structure your interrogation more clearly”, “Remember to disinfect your hands prior to and following the procedure”, and “Clearly announce your next steps to the patient”). These could be checked by the examiners.

The resulting feedback tool was tested during the subsequent OSCE that took place in the winter term 2015/16. Examiners were asked to fill in the feedback tool during the exam. To enable the examiner to provide accurate feedback, the interval between stations was extended from one to two minutes. This additional time was needed to enable the reviewer to complete the feedback tool. The tool was then sent to the students via email after they had completed the OSCE. Students did not receive additional direct feedback, and the feedback tools were not shown to them during the OSCE.

#### Evaluation

To evaluate the feedback tool, two anonymous web-based surveys were conducted: one with the OSCE examiners and the other with the students. The surveys used a six-point Likert scale (1 = *totally agree* to 6 = *totally disagree*), with nine items for the students and four items for the examiners. In addition, both surveys asked for suggestions to improve the feedback tool. These surveys were used after both OSCEs in which the feedback tool was implemented.

#### Revision of the feedback tool

Based on the results of the surveys, the feedback tool was revised by medical education specialists (MR, JS, and TS). Therefore, the comments made by the students and examiners were analysed, and common suggestions for improvement were integrated into the tool. The resulting tool was then retested during the subsequent OSCE. Afterwards, students and examiners evaluated the tool in the same manner as previously. Students had the opportunity to request to speak personally to the examiners if they had further questions after they received their feedback.

### Data analysis

Data were analysed using Excel (Microsoft Office Excel 2007,©Microsoft Corporation). Continuous variables were represented as the mean and its standard deviation. Categorical variables were described as frequencies and percentages.

## Results

During the OSCE in which the first feedback tool was used (winter term 2015/16), 150 students and 24 examiners participated, and all of them agreed to participate in the present study. The average age of the students was 24.9 ± 3.0 years, and they had been studying medicine for an average of 3.9 ± 1.2 years. Approximately 62.1% of them were female. Sixty-eight students answered the questionnaire (response rate 45.3%), as did 15 examiners (response rate 62.5%). Table 1 shows the sociodemographic data of the examiners.

**Table 1:** Characteristics of the examiners participating in the study in the first and second OSCEs

	First OSCE	Second OSCE
Number of previous OSCEs as examiner	3.6 ± 2.2	3.8 ± 2.5
**Gender**		
Female	21.4%	30.8%
Male	78.6%	69.2%
**Surgical discipline**		
Thoracic and heart surgery	7.1%	15.3%
Oral, cranio-maxillofacial, and facial plastic surgery	14.2%	7.7%
Vascular surgery	28.6%	23.1%
General and visceral surgery	35.7%	30.8%
Trauma surgery	21.4%	23.1%
**Stage of advanced professional training**		
Resident	38.5%	69.2%
Consultant	15.4%	0%
Senior physician	46.2%	30.8%
Chief physician	7.9%	0%

Shown as the mean + standard deviation

During the OSCE in which the revised feedback tool was implemented (summer term 2016), 201 students and 27 examiners participated. The average age was 23.7 years old, and the students had been studying medicine for 3.6 ± 0.7 years. In total, 59.7% of them were female. Seventy-eight students answered the questionnaire after the second OSCE (response rate 38.8%), as did 13 examiners (response rate 48.1%). Table 1 shows the sociodemographic data of the study participants.

### Feedback tool

**Fig. 2:**
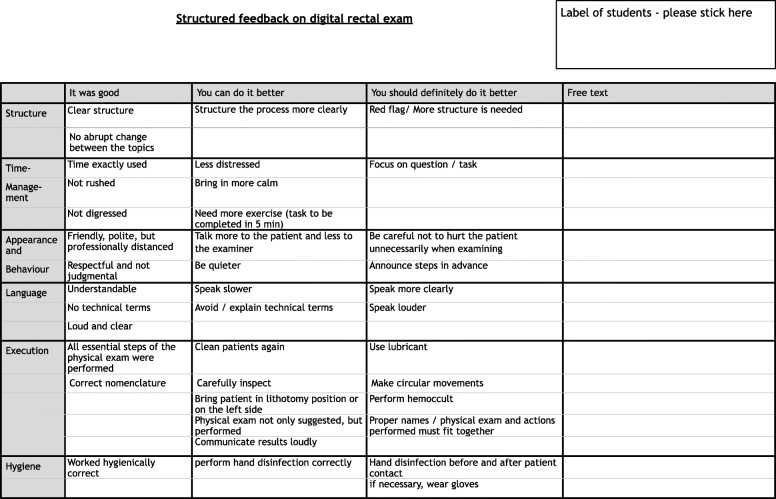
Example of the feedback tool

To meet the students’ expectations and need for individual and specific feedback, a baseline was created for use at all OSCE stations (e.g., taking a patient’s history, physical examination, practical skills, and obtaining informed consent for a surgical operation). This was supplemented with station-specific items (e.g., explaining special risks for an intervention at OSCE stations that required informed consent). During the revision of the feedback tool, often-made free-text comments were added as items for the preformulated options. Each of these items was rated on the three-point scale presented above (“You performed well in …” , “You could improve in …” , and “You definitely need to improve in …)” . In addition to the preformulated options, each domain had a separate column for individualized comments. Figure 2 shows one example of the feedback tool.

### Students’ evaluation of the resulting feedback tool

**Fig. 3:**
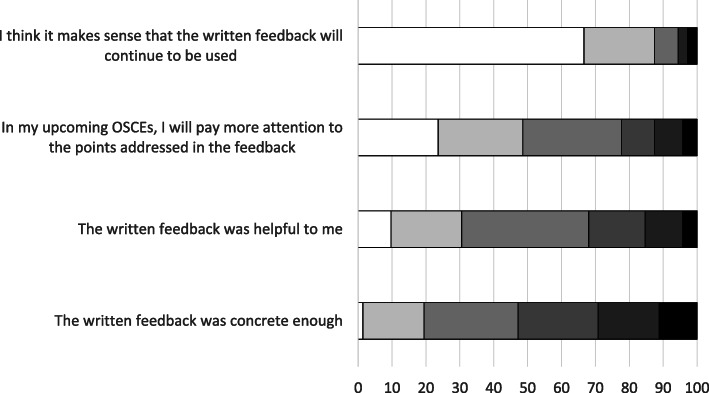
Result of the students’ evaluation. Shown as percent;  total agree;  mostly agree;  rather agree;  rather disagree;  mostly disagree;  totally disagree

Seventy-eight students answered the questionnaire (response rate 38.8%). In total, 87.5% of these students *agreed* or *rather agreed* that written feedback should continue to be used in future OSCEs. However, over 50% of the students pointed out that the feedback was still not concrete enough. Figure 3 shows the results of the students’ evaluation, and Table 2 shows the free-text comments that the students made during the evaluation.

**Table 2:** Free-text comments made by the students during the evaluation

Strengths	Suggestions for improvement
Personally matched. Mistakes can be better understood, and the examiner’s impression of one is also more comprehensible.	Unfortunately, it is still not detailed enough. Provide the OSCE checklists in a reduced form to let the students know what is REALLY expected. (...) If you want to learn something from the OSCE for life, the feedback is still not transparent.
It is good that feedback is generally introduced. It is good that each station is evaluated individually and that the feedback addresses station-specific points.	More comments. Some examiners wrote comments; these were sometimes much more helpful than crosses on the formulated sheet or more specific. It would be nice if more examiners wrote comments.
Good supplement to pure grading. A weakness in my dialogue with patients became very clear to me.	Written comments from the examiner help better than circling the pre-formulated statements.
I am now more aware of the impression I leave on the examiner during the examination. This is very helpful. The topics discussed are rationally selected.	The examiners should write more comments. Some examiners have done that - but there was not even enough space for the comments; however, other examiners commented nothing, which I thought was a pity.
To get any feedback about what you did wrong or right in the examination. Without this feedback about what you did right or wrong, it is not possible to improve. In addition, then, in my opinion, the whole exam did not make much sense!	It would have to be more detailed, not just the tick on the feedback tool. The best would be direct personal feedback after each exam!

### Examiners’ evaluation of the resulting feedback tool

**Fig. 4:**
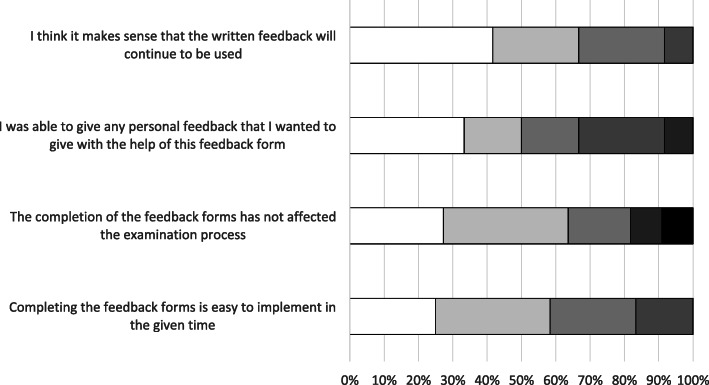
Result of the examiners’ evaluation. Shown as percent;  total agree;  mostly agree;  rather agree;  rather disagree;  mostly disagree;  totally disagree

Thirteen examiners answered the questionnaire (response rate 48.1%). In total, 91.6% of them stated that written feedback should be continued and agreed that they were able to “give any personal feedback that I wanted to give with the help of this feedback form”. However, over one-quarter of the examiners said that filling out the feedback tools had affected the examination itself. The results of the examiners’ evaluation are shown in Fig. 4, while Table 3 shows the free-text comments that the examiners made during the evaluation.

**Table 3:** Free-text comments made by the examiners during the evaluation

Strengths	Suggestions for improvement
Ability to teach students their strengths and weaknesses with relatively little effort.	More time for individual rating.
Possibility to show the students their outside impact and to assign them to structural strengths and weaknesses.	More structured feedback sheet that can be fulfilled faster.
Individual.	

### Cost analysis

Because the creation of each feedback tool takes approximately 1 h and an individual questionnaire must be created for each station, 80 OSCE stations and a student assistant’s salary of €13.50 per hour would result in a one-time financial cost of approximately €1000 for the initial implementation.

After the first implementation, the time required is approximately 5 min per participating student, as the completed feedback tools must be viewed, scanned, and sent via email. Based on this, the cost for 150 students is approximately €200 per semester.

## Discussion

In the present study, we were able to demonstrate that the implementation of written structured feedback into a curricular summative OSCE in surgery was possible within the given requirements appreciated by students and examiners.

Although the students stated that the feedback tool should continue to be used in upcoming OSCEs, they wanted even more individualized and concrete feedback. One way to create more individualized feedback, while still allowing the students to review it after the examination, is addressed by Harrison et al., who implemented oral feedback into an OSCE via an audio recording provided to the students [23]. Though the students rated this feedback positively, the authors noted that it was not standardisable. Wardman et al. also compared written feedback to audio-recorded feedback. In their study, individualized written feedback was compared to oral feedback regarding the general performance of the student cohort at each station. Neither method included predefined structuring [17]. In contrast, we developed a method to provide feedback in which the thematic focal points were predefined.

To meet the students’ desire for more individualized feedback without a loss of standardization, we decided to allow free-text comments by the examiners in each domain. These comments were more valuable to the students than were the preformulated answers. However, because the OSCE is already associated with a high mental workload for the examiners [24], it is important to simplify the feedback process by providing examiners with preformulated answers. Furthermore, the feedback tool ensures that the largest part of the feedback is legible regardless of the examiner’s handwriting, which is shown to be important for students [25]. Additionally, Wardman et al. found that 35% of the examiners stated that they needed more time to write feedback [17]. Similar results were found in the present study; one-quarter of the examiners stated that completing the feedback tool had affected the OSCE itself. Nevertheless, most of the examiners agreed or rather agreed that filling out the feedback tool was easy to complete in the given time. Taking this together, there must be other reasons beyond the time used to complete the tool that could affect the examination itself. One possible reason is that the examiners are focusing on providing high-quality feedback rather than on mastering skills. This aspect becomes even more important, as the present feedback tool was implemented in a summative assessment that determines whether a student passes or fails. Against this background, it is necessary to carefully weigh the willingness to enable individual feedback and the demands to carry out an objective and reliable examination. The feedback tool described in the present study can fit both needs, even if it must be continuously adapted and improved. Furthermore, frequently occurring free-text comments made by reviewers should be summarized and integrated into the preformulated responses so that using the tool is easy for the examiners. At the same time, it is necessary to clearly communicate to the students why this is necessary and to create an objective OSCE examination that this feedback does not influence. By giving the students the opportunity to request a personal meeting with the examiners if they had further questions after they received their feedback, it was possible to provide individualized feedback to all students who participated in the OSCE and to reduce the number of personal conversations needed. In the curricular setting in which the OSCE took place, it would not have been possible to make this one-on-one talk possible for every student.

The present study demonstrates an economical way to provide individualized expert feedback to a large number of medical students in a curricular summative assessment. Bienstock et al. argued that feedback can be provided by anyone who can carry out a good observation of the student’s performance and who must be experienced with regard to both the content and the pedagogical aspect [26]. On the other hand, Lechermeier and Fassnacht were able to demonstrate in a comprehensive literature review that feedback is “most effective when provided by a source who disposes over high status and expertise” [27]. Nevertheless, medical experts are not always experts in didactics. The feedback tool presented in this study enables medical experts to provide high-quality feedback by using preformulated answers, even if they do not have the didactical expertise necessary to formulate this feedback.

By implementing the feedback tool, the examiners were forced to change from a largely passive approach (just rating the students’ performance on a checklist without having the opportunity to interact with the students) to a more active participation. Because of this, they had to think more about the performance shown by the students as a whole and about the specific feedback they wanted to give based on this. As shown by previous studies in medical [28] and nonmedical [29] education, participation in an examination leads to reflection on the examiners’ own teaching. By using the feedback tool, this effect was reinforced.

Some limitations should be discussed. On the one hand, the implementation was performed and evaluated at a single medical school and for a surgical OSCE. Further studies should evaluate transferability to other subjects and other schools or academic areas. In addition, the study did not analyse the impact of this feedback on further examinations or real-life practices. However, due to the proven effectiveness of feedback in many other areas of medical education [2, 30–32], it can be assumed that this feedback has a positive impact. Nevertheless, further studies should analyse whether the feedback given in the present study impacts students’ learning. Therefore, it seems necessary to analyse how and how often students used the feedback.

Another limitation is the small response rate (38.8% for students and 48.1% for evaluators). Therefore, it seems possible that selection bias may have influenced the results. It is conceivable that only those students and examiners who already had a positive attitude towards feedback participated in the survey and, thus, that the value of their feedback was overestimated. On the other hand, a way to provide feedback to a large number of students during a summative assessment was implemented. Thus, it was possible to create and analyse the feedback tool under ‘in vivo’ conditions and not only in a defined experimental setting. Because of this, transferability of this method to other medical schools and further curricular examinations is likely.

## Conclusion

The implementation of structured, written feedback in a curricular summative examination is feasible, and students and examiners would like such feedback to be constant.

## Data Availability

The datasets used and analysed during the current study are available from the corresponding author upon reasonable request.

## Abbreviation

OSCE: Objective structured clinical examination

## References

[1] Eraut M (2006). Feedback. Learn Health Soc Care.

[2] Clynes MP, Raftery SE (2008). Feedback: an essential element of student learning in clinical practice. Nurse Educ Pract.

[3] Parboteeah S, Anwar M (2009). Thematic analysis of written assignment feedback: implications for nurse education. Nurse Educ Today.

[4] Nesbitt A, Pitcher A, James L, Sturrock A, Griffin A (2014). Written feedback on supervised learning events. Clin Teach.

[5] van de Ridder JM, Stokking KM, McGaghie WC, ten Cate OT (2008). What is feedback in clinical education?. Med Educ.

[6] Ramaprasad A (1983). On the definition of feedback. Behav Sci.

[7] Russeler M, Schill A, Kalozoumi-Paisi P, Ganzert C, Arheilger L, Sterz J (2017). Teaching in perspective - how medical students assess their practical clinical training in surgery. Zentralbl Chir.

[8] Beckman TJ (2004). Lessons learned from a peer review of bedside teaching. Acad Med.

[9] Mensink PJ, King K (2020). Student access of online feedback is modified by the availability of assessment marks, gender and academic performance. Br J Educ Technol.

[10] Henry D, Vesel T, Boscardin C, van Schaik S (2018). Motivation for feedback-seeking among pediatric residents: a mixed methods study. BMC med educ.

[11] Jünger J, Schellberg D, Nikendei C (2006). Student ´s self-assessment of clinical competence and objective clinical performance in OSCE evaluation. GMS Z Med Ausbild.

[12] Sinclair HK, Cleland JA (2007). Undergraduate medical students: who seeks formative feedback?. Med Educ.

[13] Harden RM, Stevenson M, Downie WW, Wilson G (1975). Assessment of clinical competence using objective structured examination. Br Med J.

[14] Müller S, Dahmen U, Settmacher U (2016). Application of the objective structured clinical examination (OSCE) in German medical schools: an inventory. Gesundheitswesen..

[15] Harrison CJ, Konings KD, Schuwirth L, Wass V, van der Vleuten C (2015). Barriers to the uptake and use of feedback in the context of summative assessment. Adv Health Sci Educ Theory Pract.

[16] Humphrey-Murto S, Mihok M, Pugh D, Touchie C, Halman S, Wood TJ (2016). Feedback in the OSCE: what do residents remember?. Teach Learn Med.

[17] Wardman MJ, Yorke VC, Hallam JL (2018). Evaluation of a multi-methods approach to the collection and dissemination of feedback on OSCE performance in dental education. Eur J Dent Educ.

[18] Morris C, Chikwa G (2016). Audio versus written feedback: exploring learners’ preference and the impact of feedback format on students’ academic performance. Act Learn High Educ.

[19] Haghani F, Hatef Khorami M, Fakhari M (2016). Effects of structured written feedback by cards on medical students' performance at mini clinical evaluation exercise (mini-CEX) in an outpatient clinic. J Adv Med Educ Prof.

[20] Junod Perron N, Louis-Simonet M, Cerutti B, Pfarrwaller E, Sommer J, Nendaz M. Feedback in formative OSCEs: comparison between direct observation and video-based formats. Med Educ Online. 2016;21. 10.3402/meo.v21.32160. 10.3402/meo.v21.32160 PMC5103667 27834170

[21] Newton PM, Wallace MJ, McKimm J (2012). Improved quality and quantity of written feedback is associated with a structured feedback proforma. J Educ Eval Health Prof.

[22] Russeler M, Weber R, Braunbeck A, Flaig W (2010). Lehrteam des Zentrum C, Marzi I et al. [training of practical clinical skills in surgery - a training concept for medical students]. Zentralbl Chir.

[23] Harrison CJ, Molyneux AJ, Blackwell S, Wass VJ (2015). How we give personalised audio feedback after summative OSCEs. Med teach.

[24] Byrne A, Soskova T, Dawkins J, Coombes L (2016). A pilot study of marking accuracy and mental workload as measures of OSCE examiner performance. BMC med educ.

[25] Hepplestone S, Chikwa G (2014). Understanding how students process and use feedback to support their learning. Pract Res High Educ.

[26] Bienstock JL, Katz NT, Cox SM, Hueppchen N, Erickson S, Puscheck EE (2007). Association of Professors of Gynecology and Obstetrics undergraduate medical education committee. To the point: medical education reviews--providing feedback. Am J Obstet Gynecol.

[27] Lechermeier J, Fassnacht M (2018). How do performance feedback characteristics influence recipients’ reactions? A state-of-the-art review on feedback source, timing, and valence effects. Manag Rev Quarterly.

[28] Sterz J, Bender B, Linßen S, Stefanescu MC, Hoefer SH, Walcher F, Voss J, Seifert LB, Ruesseler M (2019). Effects and consequences of being an OSCE examiner in surgery-a qualitative study. J Surg Educ.

[29] Ní Chróinín D, Cosgrave C (2013). Implementing formative assessment in primary physical education: teacher perspectives and experiences. Phys Educ Sport Pedagog.

[30] Muessig M, Sterz J, Stefanescu M-C, Bender B, Hoefer SH, Ruesseler M. The Impact of Video Feedback on Acquiring Competency in Basic Surgical Skills (Sterile Working) in an Undergraduate Medical Training Program: A Comparative Effectiveness Analysis. J Advanc Educ Res. 2017;2(3). 10.2196/24043.

[31] Naik ND, Abbott EF, Gas BL, Murphy BL, Farley DR, Cook DA (2018). Personalized video feedback improves suturing skills of incoming general surgery trainees. Surgery..

[32] Rammell J, Matthan J, Gray M, Bookless LR, Nesbitt CI, Rodham P, et al. Asynchronous unsupervised video-enhanced feedback as effective as direct expert feedback in the long-term retention of practical clinical skills: randomised trial comparing 2 feedback methods in a cohort of novice medical students. J surg educ. 2018. 10.1016/j.jsurg.2018.03.013. 10.1016/j.jsurg.2018.03.013 29748142

